# *lncRNA-NRF* is a Potential Biomarker of Heart Failure After Acute Myocardial Infarction

**DOI:** 10.1007/s12265-020-10029-0

**Published:** 2020-05-21

**Authors:** Li Yan, Yu Zhang, Wei Zhang, Sheng-Qiong Deng, Zhi-Ru Ge

**Affiliations:** 1 Ningxia Medical University, Yinchuan, 750000 China; 2 Department of Cardiology, Shanghai Gongli Hospital, The Second Military Medical University, Shanghai, 200135 China; 3 Department of Emergency Medicine, Shanghai Gongli Hospital, The Second Military Medical University, Shanghai, 200135 China; 4 Department of Clinical Laboratory, Shanghai Gongli Hospital, The Second Military Medical University, Shanghai, 200135 China

**Keywords:** Heart failure, Acute myocardial infarction, lncRNA-NRF, Diagnosis

## Abstract

Long non-coding RNAs (lncRNAs) are a new focus in cardiovascular diseases. The necrosis-related factor (NRF) is a newly discovered lncRNA, which is increased in myocardial injury. We investigated the role of lncRNA-NRF in heart failure (HF) after acute myocardial infarction (AMI) to find a biomarker for early HF detection. This was a cross-sectional study of 76 AMI patients with HF and 58 AMI patients without HF. lncRNA-NRF was shown to be increased in AMI patients with HF compared with AMI patients without HF and had predictive value for diagnosis of HF. It had a high diagnostic value for HF (AUC, 0.975), while the AUC for N-terminal pro-brain natriuretic peptide was 0.720. Our findings suggest that lncRNA-NRF may represent a marker of risk for development of HF post-AMI.

**Electronic supplementary material:**

The online version of this article (10.1007/s12265-020-10029-0) contains supplementary material, which is available to authorized users.

## Introduction

Acute myocardial infarction (AMI), one of the most serious coronary artery diseases [1], and the heart failure (HF) that often follows are among the leading causes of death and disability worldwide [2–4]. One contributing factor is the existence of “myocardial reperfusion injuries” [4], whereby myocardial injury and cardiomyocyte death occur on the reperfusion of acutely ischemic myocardium because of a number of factors [5–7], including mitochondrial calcium overload, mitochondrial oxidative stress, ATP depletion [5, 7, 8], and the opening of the mitochondrial permeability transition pore [6]. Medical therapy is the most effective treatment, but a lack of symptoms in the early stages leads to poor prognosis. Therefore, exploring effective biomarkers is of great importance for early detection and improving prognosis in HF after AMI.

Long non-coding RNAs (lncRNAs) are located in the cell nucleus or cytoplasm and have a length of more than 200 nucleotides [9, 10]. Recently, studies have demonstrated that lncRNAs are involved in many complex cell processes in cardiovascular disease [11–13]. For example, the lncRNA necrosis-related factor (NRF) was confirmed to be increased in myocardial injury, and silencing of NRF increased miR-873 expression and decreased levels of the miR-873 targets, receptor-interacting serine/threonine-protein kinase 1 (RIPK1) and RIPK3, which led to a sharp reduction in myocardial necrosis [14]. However, the role of lncRNA-NRF in the diagnosis of HF after AMI remains unclear. Here, we assessed the expression of lncRNA-NRF in patients with AMI and identified the diagnostic value of lncRNA-NRF for HF in AMI patients.

## Materials and methods

### Participants

The study was designed as a cross-sectional study and was conducted between June 2018 and February 2020 in the Department of Cardiology of Shanghai Gong Li Hospital. Patients with acute HF caused by serious arrhythmias, acute valve dysfunction, or other reasons, and those with chronic HF, pregnancy, cancer, acute infectious diseases, hepatic dysfunction, or abnormal renal function were excluded. A final population of 248 participants was enrolled in the study, and all patients who were diagnosed with AMI underwent percutaneous coronary intervention (PCI) as soon as possible. Based on diagnostic standards, the participants with HF post-PCI were divided into two groups of HF after AMI (*n* = 76) and non-HF after AMI (*n* = 58), and 30 basic clinical data-matched controls, 39 nonischemic HF patients, and 45 stable CAD patients. The AMI diagnosis was based on the 2017 ESC Guidelines for the management of AMI in patients presenting with ST-segment elevation [15]. At least one of the following was present in AMI patients: symptoms (i.e., persistent chest pain > 30 min) and signs (i.e., 12-lead electrocardiogram, ST-segment elevation > 0.2 mm) consistent with myocardial ischemia, at least a twofold increase in troponin I (TnI) [15]. The HF diagnosis was based on the 2016 ESC Guidelines for the diagnosis and treatment of acute and chronic HF [16]. At least one of the following was present in such patients: typical symptoms (dyspnea, fatigue), specific signs (elevated jugular venous pressure, hepatojugular reflux, pulmonary crackles, peripheral edema), age-specific levels of N-terminal pro-brain natriuretic peptide (NT-proBNP) (ng/L) (age < 50 years: > 450 ng/L, age 50–75 years: > 900 ng/L, age > 75 years: > 1800 ng/L), and left ventricular ejection fraction (LVEF) < 50% [16]. Finally, 76 patients with HF after AMI were included in the study. All participants provided written informed consent before enrollment. The study was conducted in accordance with the principles contained within the Declaration of Helsinki.

### Anthropometric measurements

Anthropometric determinations and blood extractions were performed on a single day. Height and weight were measured using calibrated portable electronic weighing scales and portable inflexible height measuring bars with barefoot participants wearing light indoor clothing. BMI was calculated using the standard BMI formula: body mass (in kilograms) divided by square of height (in meters).

### Blood sample preparation

All blood samples were collected after participants had fasted overnight. Clinical variables included TnI, myoglobin, creatine kinase-MB, total cholesterol (TC), triglyceride (TG), low-density lipoprotein (LDL), high-density lipoprotein (HDL), serum creatinine (SCr), cystatin, urea nitrogen, and estimated glomerular filtration rate (eGFR). Serum TnI, CK-Mb, TG, TC, LDL-C, and HDL-C levels and other routine serum biochemical parameters were measured using a biochemical analyzer (Ortho-3160; Pencoed, Bridgend, UK). Levels of NT-proBNP and lncRNA-NRF were measured at immediate diagnosis (before PCI), intraprocedural, and postoperatively at 4 h, 12 h, 24 h, and 72 h. Serum NT-proBNP was measured using a biochemical analyzer (Nano-Checker710; Nano-Ditech, Grandbury, American). All measurements were obtained using blinded quality control specimens at the Department of the Biochemical Laboratory at Shanghai Gong Li Hospital. Venous blood was obtained from 30 basic clinical data-matched controls, 39 HF patients, and 45 CAD patients. All blood samples for the lncRNA-NRF analysis were immediately placed into blood collection tubes containing EDTA, and were stored at − 80 °C before RNA extraction. The expression of lncRNA-NRF was detected through the RT-PCR analysis.

RNA extraction and RT-PCR analysis

Total RNA was isolated from venous blood samples using Trizol reagent (Invitrogen, Carlsbad, USA), and RNA quantification was performed using a Nanodrop (Thermo Scientific, Rockford, IL, USA). cDNA was synthesized using an Improm II reverse transcription kit (Promega, Madison, WI, USA) following the manufacturer’s instructions. RT-PCR was performed using GAPDH as an internal control and SYBR Green to detect lncRNA-NRF levels, which were quantified through the 2^−ΔΔCt^ method. The primers for the target genes (Table 1) were obtained from GenePharma (Shanghai, China).

**Table 1:** Anthropometric and biochemical characteristics of the subjects

	Control	HF post-AMI	Non-HF post-AMI	HF	CAD	*P* value
Male (*n*, %)	13 (43)	42 (55)	33 (57)	19 (49)	22 (49)	0.712
Age (years)	59.63. ± 10.39	63.36 ± 7.29	62.84 ± 11.12	61.69 ± 7.18	64.36 ± 7.68	0.189
BMI (kg/m2 )	24.49 ± 3.16	23.91 ± 2.76	23.77 ± 4.08	24.05 ± 4.04	26.06 ± 4.72	0.019
Smoking history (*n*, %)	10 (33)	43 (57)	22 (38)	10 (26)	13 (29)	0.060
Diabetic (*n*, %)	9 (30)	30 (39)	17 (28)	11 (28)	14 (31)	0.663
Hypertensive (*n*, %)	22 (73)	54 (71)	41 (71)	28 (72)	35 (78)	0.935
TG (mmol/L)	1.43 ± 0.71	1.36 ± 1.10	1.57 ± 0.75	1.07 ± 0.54	1.68 ± 0.92	0.016
TC (mmol/L)	4.41 ± 1.16	4.27 ± 1.04	4.50 ± 1.07	4.01 ± 1.10	4.42 ± 1.50	0.315
LDL-C (mmol/L)	2.70 ± 0.95	2.78 ± 0.93	2.97 ± 0.88	2.41 ± 1.00	2.68 ± 1.37	0.127
HDL-C (mmol/L)	1.08 ± 0.23	1.01 ± 0.27	1.00 ± 0.45	1.09 ± 0.39	1.04 ± 0.34	0.642
SCr (umol/L)	68 (52, 86)	73 (60, 91)	66 (50, 80)	94 (79, 119)	68 (59, 79)	0.313
UN (umol/L)	0.90 (0.74, 1.12)	5.3 (3.98, 6.89)	4.66 (3.86, 5.99)	7.83 (4.57, 10.34)	5.15 (4.22, 6.22)	0.806
CysC (mg/L)	5.22 (4.16, 7.44)	1.11 (0.93, 1.37)	0.81 (0.74, 1.04)	1.17 (0.96, 1.50)	0.93 (0.81, 1.14)	<0.01**
eGFR (mL/min/1.73 m^2^)	90 (67, 96)	89 (67, 98)	94 (84.5, 102)	75 (48, 88)	89 (82, 98)	0.112
LVEF (%)	60.83 ± 6.86	55.59 ± 8.71	61.46 ± 5.27	54.61 ± 8.53	64.01 ± 6.25	< 0.001***
LVFS (%)	32.63 ± 4.76	29.31 ± 5.55	33.03 ± 3.75	29.01 ± 5.89	35.13 ± 4.75	< 0.001***
LVEDD (mm)	47.47 ± 5.15	47.89 ± 5.74	47.45 ± 4.66	52.03 ± 8.98	47.44 ± 4.90	<0.01**
Postoperative NT-proBNP (ng/L)	41 (20, 100)	2136 (1487, 3461)	249 (151.5, 442)	2645 (1903, 3678)	120 (20, 378)	< 0.001***
Intraprocedural NT-proBNP (ng/L)	/	478 (134, 950)	204 (20.1, 434.5)	/	/	< 0.001***
TnI	0.02 ± 0.03	18.75 ± 27.71	11.28 ± 21.94	0.03 ± 0.02	0.26 ± 1.30	< 0.001***
TIMI	/	2.66 ± 0.88	2.64 ± 0.77	/	/	0.822
Number of branch lesions	/	1.01 ± 1.37	1.17 ± 1.40	/	/	0.511
Immediate diagnosis of lncRNA-NRF	0.95 (0.87, 1.02)	1.71 (1.21, 2.02)	0.56 (0.48, 0.61)	1.07 (0.93, 1.29)	0.67 (0.59, 0.86)	< 0.001***
Intraprocedural of lncRNA-NRF	/	1.77 (1.32, 3.50)	1.01 (0.83, 1.14)	/	/	< 0.001***
Postoperative 4 h of lncRNA-NRF	/	1.18 (0.87, 1.63)	0.66 (0.56, 0.74)	/	/	< 0.001***
Postoperative 12 h of lncRNA-NRF	/	1.34 (0.93, 1.52)	0.83 (0.65, 1.00)	/	/	< 0.001***
Postoperative 24 h of lncRNA-NRF	/	1.07 (0.98, 1.47)	0.98 (0.86, 1.06)	/	/	< 0.001***
Postoperative 72 h of lncRNA-NRF	/	1.01 (0.89, 1.29)	0.95 (0.80, 1.04)	/	/	<0.01**

Results are expressed as mean ± standard deviation, median (interquartile range) or *n* (%). *BMI*, body mass index; *TG*, triglycerides; *TC*, total cholesterol; *LDL-C*, low-density lipoprotein cholesterol; *HDL-C*, high-density lipoprotein cholesterol; *SCr*, serum creatinine; *CysC*, cystatin; *UN*, urea nitrogen; *eGFR*, estimated glomerular filtration rate; *NT-proBNP*, N-terminal pro-brain natriuretic peptide; *LVEF,* left ventricular ejection fraction; *LVFS*, left ventricular fractional shortening; *LVEDD*, left ventricular end-diastolic dimension’ TnI, troponin I; *TIMI*, thrombolysis in myocardial infarction

*P < 0.05, **P < 0.01, ***P < 0.001

## Statistical analysis

Continuous variables are expressed as mean ± standard deviations or median (interquartile range) and categorical variables as numeral (percentage). Independent Student’s *t* tests or one-way ANOVA for normal distribution and Wilcoxon rank-sum tests for asymmetric distribution were used to analyze the differences in continuous variables. Chi-squared tests and Fisher’s exact tests were used to analyze categorical variables. The association between lncRNA-NRF and serum NT-proBNP was determined using Pearson correlation coefficient. The diagnostic value of lncRNA-NRF was estimated through building receiver operating characteristic (ROC) curves. A *P* value < 0.05 was considered statistically significant.

## Results

### Baseline clinical characteristics of the study population

This study included 76 AMI patients with HF post-PCI and 58 AMI patients without HF. The clinical characteristics of the study population are shown in Table 1. There were no differences in age (*P* = 0.189), sex (*P* = 0.712), BMI (*P* = 0.019), medical history, presence of diabetes (*P* = 0.663), hypertension (*P* = 0.935), or smoking history (*P* = 0.060) between the five groups. There were significantly higher levels of CysC (*P* = 0.001), but no significant differences in SCr (*P* = 0.313), eGFR (*P* = 0.112), and urea nitrogen (*P* = 0.806) between the five groups. Remarkably, circulating lncRNA-NRF levels were significantly elevated in the HF group compared with the non-HF group after AMI (*P* < 0.001) (Table 1).

### RT-PCR analysis of the expression of IncRNA-NRF

**Fig. 1:**
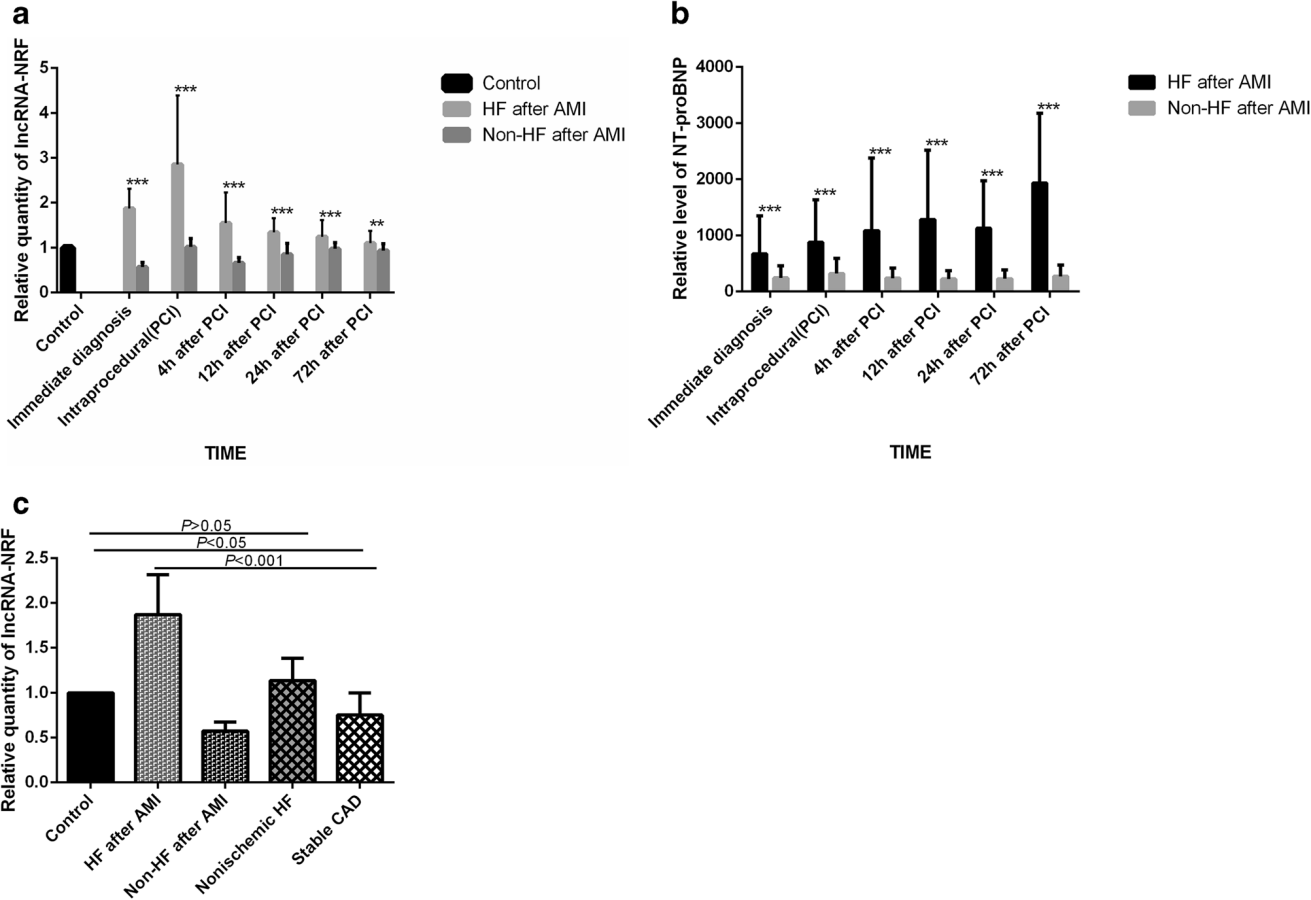
Relative average lncRNA-NRF expression in all the patients. **a** Circulating lncRNA-NRF levels in AMI patients with HF and non-HF. **b** Venous blood NT-proBNP level in AMI patients with HF and non-HF. **c** Circulating lncRNA-NRF levels in heart failure and stable CAD patients and at the time of diagnosis in patients with HF after AMI and non-HF after AMI. *P < 0.05, **P < 0.01, ***P < 0.001

RT-PCR analysis was used to measure the levels of circulating lncRNA-NRF in each group. The results demonstrated that the levels of circulating lncRNA-NRF were increased in patients with HF post-PCI compared with non-HF patients, reached a peak in intraprocedural and gradually returned to base line levels at 3 days (Fig. 1a, *P* < 0.001). However, the levels of venous NT-proBNP were increased at 3 days after PCI in patients with HF post-PCI compared with controls (Fig. 1b, *P* < 0.001). Circulating lncRNA-NRF levels expression showed higher in heart failure patients (Fig. 1c, *P* = 0.128), and decreased in patients with stable CAD. (Fig. 1c, *P* < 0.05).

### Pearson correlation coefficient of correlation between lncRNA-NRF and clinicopathological characteristics

**Fig. 2:**
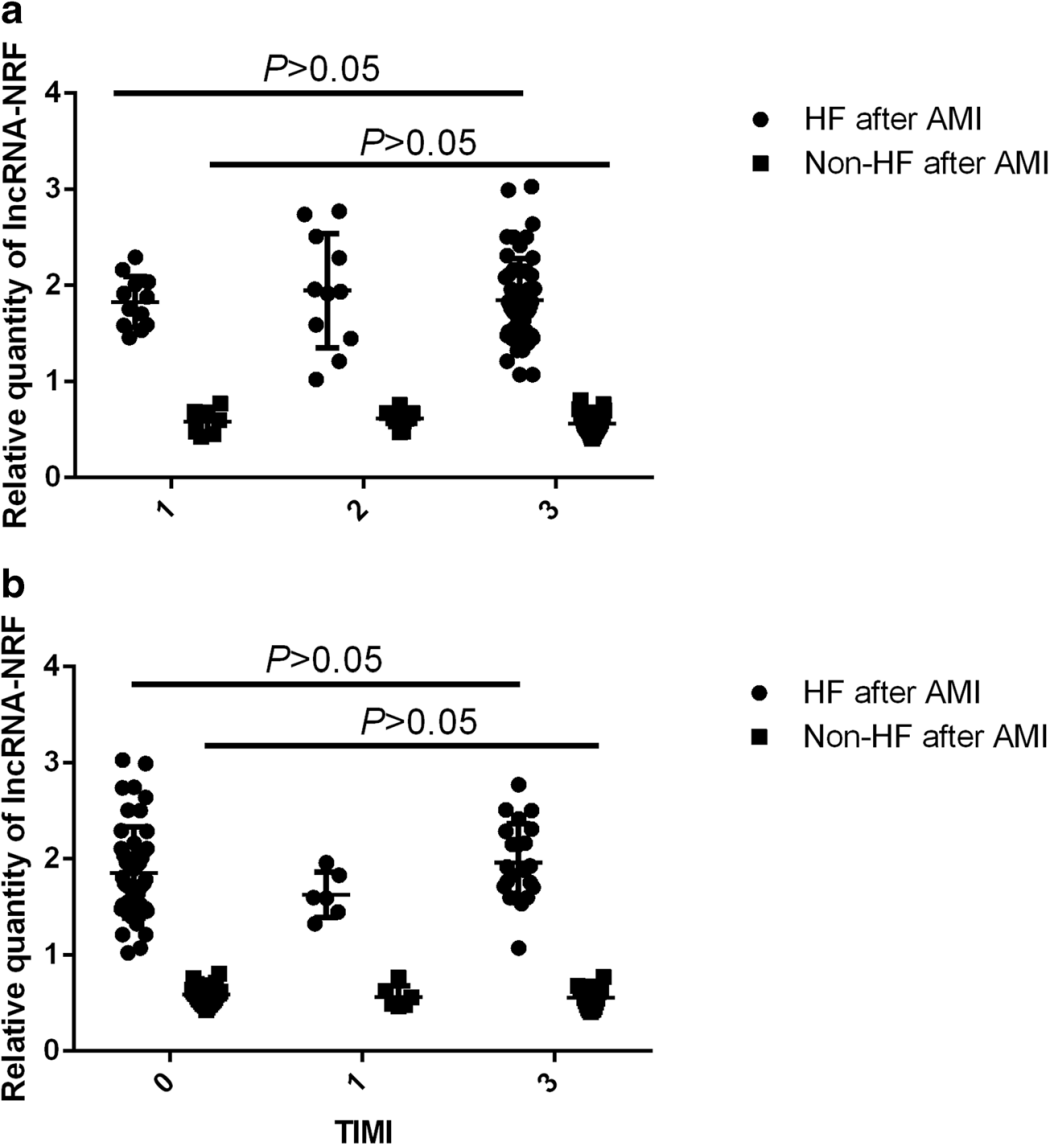
Relationship between lncRNA-NRF levels and coronary disease burden at intraprocedural timepoint. **a** LncRNA-NRF expression levels in AMI patients with different coronary lesions. **b** LncRNA-NRF expression levels in AMI patients with different TIMI flow

**Fig. 3:**
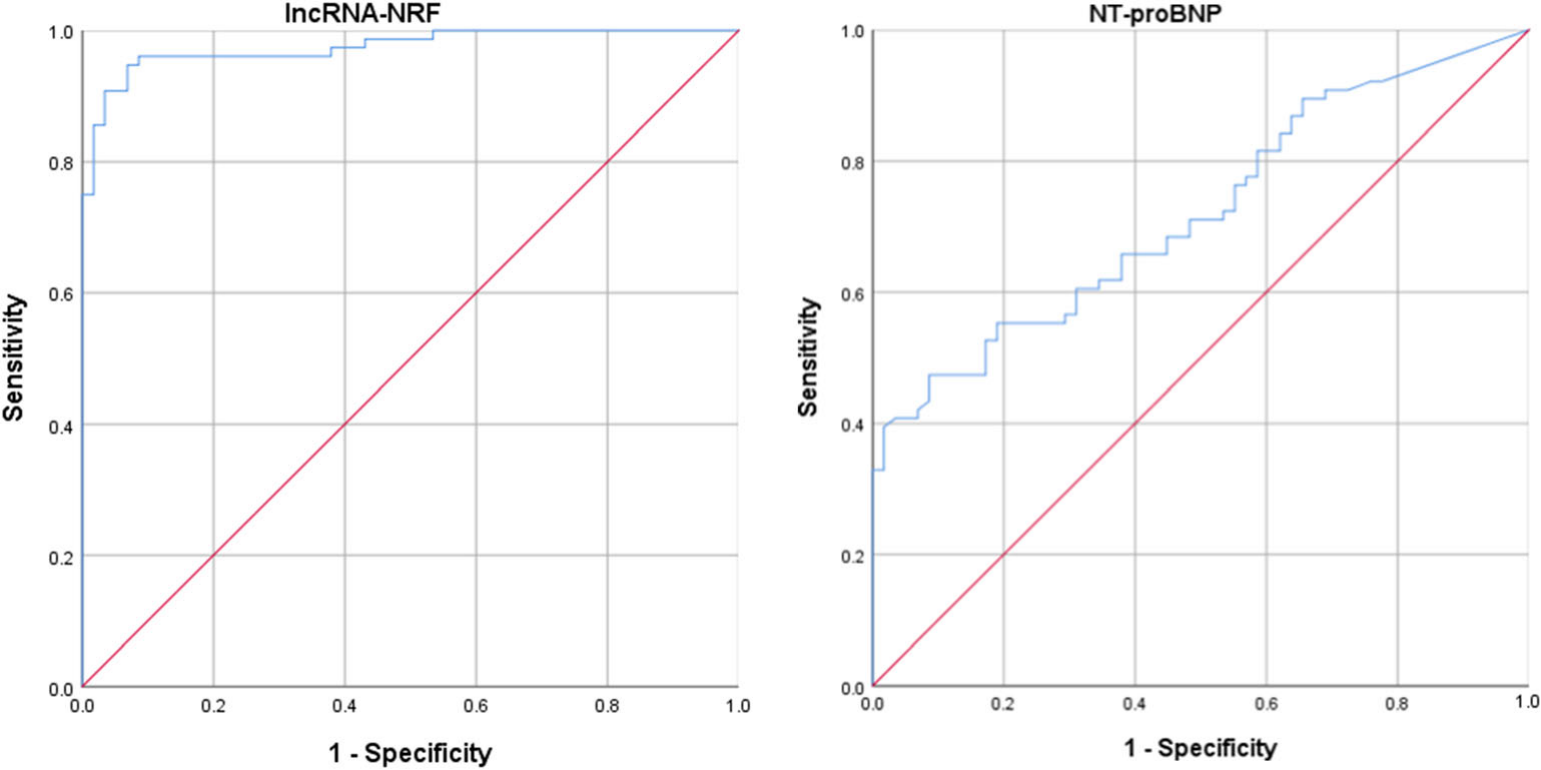
ROC curve for patients with HF after AMI based on the expression of lncRNA-NRF and NT-proBNP at the time of diagnosis

Associations between circulating lncRNA-NRF and serum NT-proBNP were examined using Pearson correlation coefficient. As shown in Table 2, circulating lncRNA-NRF levels were positively associated with serum NT-proBNP (*r*^2^ = 0.775, *P* < 0.001) and TnI (*r*^2^ = 0.062, *P* < 0.01) levels but negatively associated with LVEF (*r*^2^ = 0.225, *P* < 0.001) at the time of diagnosis. No associations with CK-Mb (*r*^2^ = 0.026, *P* = 0.074) levels were observed. Moreover, subgroup analysis found that circulating lncRNA-NRF at the time of intraprocedural was not associated with coronary disease burden or TIMI flow (Table 1; Fig. 2; *P* > 0.05) (Fig. 3).

**Table 2:** Pearson correlation coefficient of correlations between lncRNA-NRF and clinicopathological characteristics

	NT-proBNP		LVEF		TnI		CK-Mb	
	OR (95%CI)	*P* value	OR (95% CI)	*P* value	OR (95% CI)	*P* value	OR (95% CI)	*P* value
lncRNA-NRF	*r*^2^ = 0.775	< 0.001***	*r*^2^ = 0.225	< 0.001***	*r*^2^ = 0.062	< 0.01**	*r*^2^ = 0.026	0.074

Relationship between lncRNA-NRF levels and clinically relevant factors at the time of diagnosis. *NT-proBNP*, N-terminal pro-brain natriuretic peptide; *LVEF*, left ventricular ejection fraction; *TnI*, troponin I; *CK-Mb*, creatine kinase-Mb

*P < 0.05, **P < 0.01, ***P < 0.001

### ROC curve analysis of the diagnostic value of lncRNA-NRF in HF after AMI

Having established that lncRNA-NRF is present in the peripheral circulation and its levels are abnormally altered in HF patients after AMI, we sought to determine the potential utility of circulating lncRNA-NRF as a diagnostic biomarker for HF after AMI. To this end, ROC analysis was performed to evaluate the predictive power of circulating lncRNA-NRF at the time of diagnosis for HF after AMI. Our results showed that the area under the ROC curve was 0.975 (95% CI = 0.952–0.998) for lncRNA-NRF, and 0.720 (95% CI = 0.636–0.805) for NT-proBNP alone (Fig. 3).

## Discussion

The development of heart failure is common during hospitalization in a setting of acute myocardial infarction, and this risk of death is greater than that of AMI alone [17, 18]. In contrast, the incidence characteristics of HF occurring after AMI is not known. Although advances in the management of AMI, including primary PCI strategies and sustained use of evidence-based treatments, are likely to have a beneficial impact on the apparent decline in mortality, post-AMI patients that survive AMI often develop ischemic HF [19]. Therefore, identifying biomarkers that predict the high risk of HF development in these patients post-AMI is needed to optimize treatment strategies to improve their long-term prognosis. To date, several biomarkers including NT-proBNP and troponin were reported to predict cardiovascular complications after AMI; nevertheless, whether these biomarkers predict future HF in patients after AMI is unclear, and their use is limited by the heterogeneity of genetic backgrounds, environment, biological behavior, and other diseases [20]. Accumulating evidence has shown that aberrantly regulated lncRNA is correlated with the progression of various diseases, like cardiovascular disease [21–23]. Although little is known about the origin and function of lncRNAs in circulation, their sensitive and stable differential expression in the blood of patients with cardiovascular diseases and healthy people makes them a potential biomarker [24]. The mechanism of action is likely that cardiac tissue damage leads to an additional release of lncRNAs, similar to the release of proteins. The progression to HF after AMI is primarily related to left ventricular remodeling, which is a heterogeneous process influenced by multiple factors, including microvascular dysfunction, infarct size, anterior infarct location, transmural extent of necrosis, and the perfusional status of the infarct-related artery [25].

Previous studies reported many virtual lncRNAs were associated with the development of HF after AMI. For instance, NRF was closely related to the necrotic death of cardiomyocytes by acting as an endogenous RNA sponge that interacts with miR-873 in the cytoplasm [14]. Silencing of NRF increased miR-873 expression and decreased the levels of the miR-873 targets, RIPK1 and RIPK3, which led to a sharp reduction in myocardial necrosis [14]. A functional assay showed that silencing p53 reduced necrotic cell death and inhibited NRF promoter activity upon H_2_O_2_ treatment, and increased expression of miR-873 and decreased levels of RIPK1/RIPK3 were also induced by downregulated P53 levels [14]. Taken together, elucidating the regulatory relationships in the lncRNA-miRNA-mRNA axis during cardiomyocyte necrosis could shed light on potential therapeutic targets in cardiac dysfunction [12]. These results show that lncRNAs play important roles in the development and progression of HF. Substantial evidence from animal studies also suggests that NRF plays a significant role in HF [14]. However, because of a lack of clinical studies, little is known about whether the dynamic regulation of lncRNA-NRF can be viewed as a marker of disease development; however, the journey to fully explore the function of NRF in the regulation of cardiovascular physiology and pathology remains incomplete. Therefore, we concentrated on the biological behavior of LncRNA-NRF. In the current study, we first confirmed that AMI patients with HF and non-HF post-PCI had different patterns of lncRNA-NRF expression in the peripheral blood: circulating lncRNA-NRF levels in AMI patients with HF post-PCI were significantly elevated compared with non-HF after AMI controls, reached a peak in intraprocedural and gradually returned to base line levels at 3 days. (Fig. 1a, *P* < 0.01). However, the levels of venous NT-proBNP were increased at 3 days after PCI in patients with HF post-PCI compared with controls (Fig. 1b, *P* < 0.001), far behind lncRNA-NRF. Several studies have recognized a consistent association between elevated troponin levels (using regular or high-sensitivity assays) and the risk of adverse clinical outcomes among patients with heart failure and reduced EF, even in the absence of intercurrent acute coronary events [26, 27]. The present study also confirmed that circulating NRF levels are positively correlated with TnI levels and serum NT-proBNP but negatively associated with LVEF, no associations with CK-Mb levels were observed, which is consistent with the conclusions reached using the Pearson correlation coefficient. Circulating NRF levels were positively associated with the severity of HF. We also investigated the clinical significance of lncRNA-NRF. Our results suggest that abnormal lncRNA-NRF expression is related to HF diagnosis. The high diagnostic value of lncRNA-NRF was demonstrated by a ROC curve with an AUC of 0.975, and the ideal cutoff value was 0.952 (Fig. 2).

Care was taken to avoid bias in the current study. RT-PCR was performed in accordance with the manufacturer’s instructions by a trained experimenter who was unaware of the identity of the experimental groups. Moreover, in the statistical analysis, adjustments were made for the confounding effects of risk factors for HF and circulating lncRNA-NRF levels. Finally, propensity score matching was used to reduce the effects of outcome-selection bias.

The current study has some limitations. First, it is a case-control study, which means that it can only show associations, not causality. Second, all of the patients with HF and some controls were administered drugs postoperatively. The effects of medication on lncRNA-NRF levels were not investigated in the current study. Third, the present study confirmed that circulating NRF levels were positively correlated with TnI levels. However, a study limitation was that samples of troponin were only collected at the time of diagnosis and 12 h after PCI; therefore, we could not determine a correlation between serial changes in troponin and serial changes in lncRNA-NRF. Fourth, contrary to expectations, HF occurred in the majority of patients with AMI. Therefore, we should expand the sample size in the future to further study the mechanism of HF and prognosis in such patients. Fifth, because all of the study participants were Chinese, the findings may not be generalizable to other ethnicities. Our findings should be confirmed in other populations.

## Conclusions

lncRNA-NRF levels were significantly higher in Chinese post-AMI patients with HF compared with post-AMI controls. Furthermore, we demonstrated that lncRNA-NRF levels were positively associated with the severity of HF after AMI. However, the clinical application value and expression mechanism of NRF still need further study.

## Electronic supplementary materials

ESM 1 Flow chart of the inclusion of cases and controls in this study. Abbreviations: AMI, acute myocardial infraction; HF, heart failure; NT-proBNP, N-terminal pro-brain natriuretic peptide; LVEF, left ventricular ejection fraction; PCI, percutaneous coronary intervention. (PPTX)

## Abbreviations

HF: Heart failure
AMI: Myocardial infarction
BMI: Body mass index
TG: Triglycerides
TC: Total cholesterol
LDL-C: Low-density lipoprotein cholesterol
HDL-C: High-density lipoprotein cholesterol
SCr: Serum creatinine
eGFR: Estimated glomerular filtration rate
NT-proBNP: N-terminal pro-brain natriuretic peptide
LVEF: Left ventricular ejection fraction
LVFS: Left ventricular fractional shortening
LVEDD: Left ventricular end-diastolic dimension
TnI: Troponin I
TIMI: Thrombolysis in myocardial infarction
