# Supplementary material for: lncRNA-NRF is a Potential Biomarker of Heart Failure After Acute Myocardial Infarction
Source: J Cardiovasc Transl Res. 2020 May 21;13(6):1008–15. doi: 10.1007/s12265-020-10029-0 (PMC7708339; doi:10.1007/s12265-020-10029-0)
Supplement: Supplementary file 1 — Flow chart of the inclusion of cases and controls in this study. Abbreviations: AMI, acute myocardial infraction; HF, heart failure; NT-proBNP, N-terminal pro-brain natriuretic peptide; LVEF, left ventricular ejection fraction; PCI, percutaneous coronary intervention. (PPTX) [file 12265_2020_10029_MOESM1_ESM.pptx]

## Slide 1
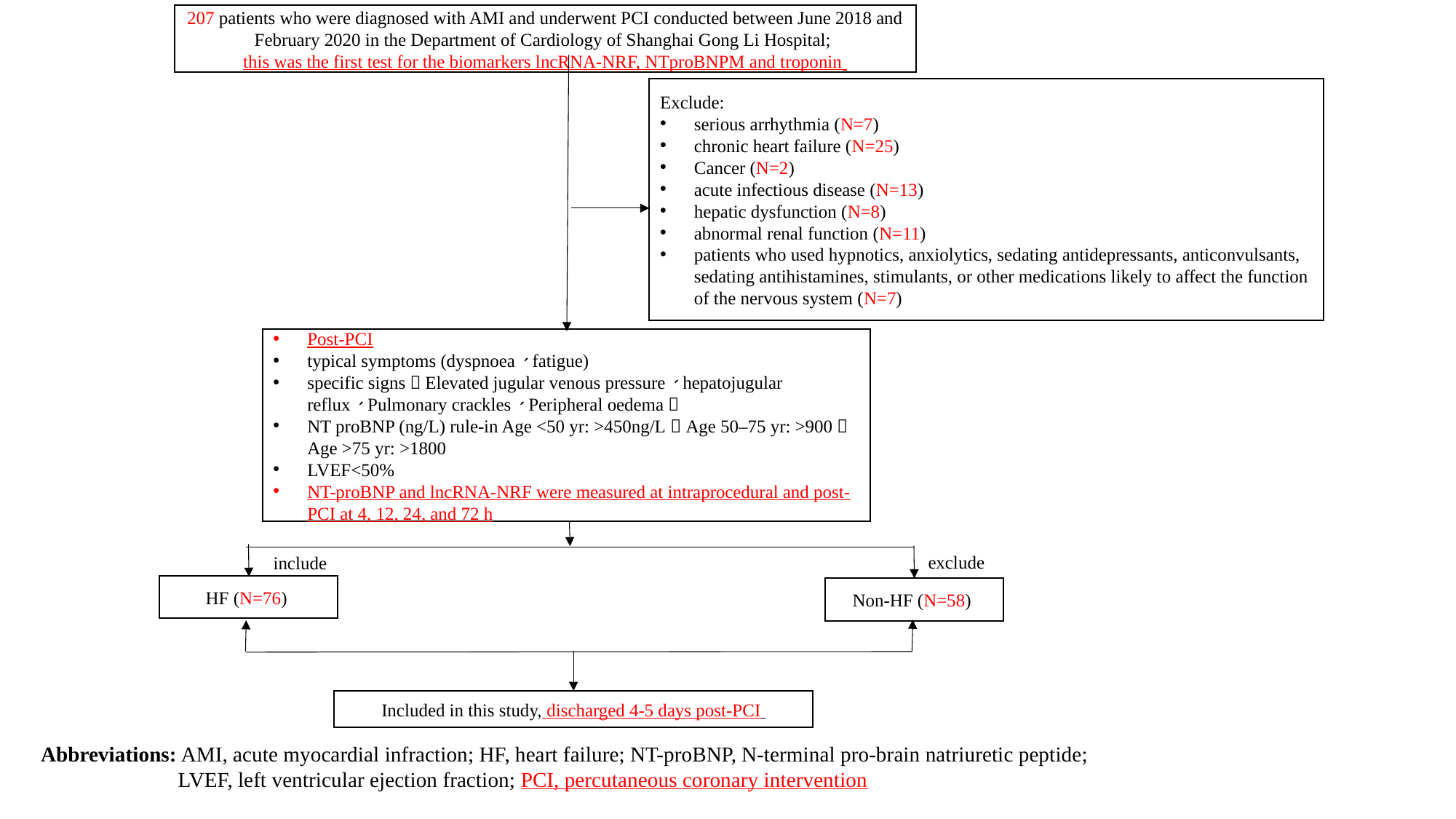

207 patients who were diagnosed with AMI and underwent PCI conducted between June 2018 and February 2020 in the Department of Cardiology of Shanghai Gong Li Hospital;
this was the first test for the biomarkers lncRNA-NRF, NTproBNPM and troponin
Non-HF (N=58)
Exclude:
serious arrhythmia (N=7)
chronic heart failure (N=25)
Cancer (N=2)
acute infectious disease (N=13)
hepatic dysfunction (N=8)
abnormal renal function (N=11)
patients who used hypnotics, anxiolytics, sedating antidepressants, anticonvulsants, sedating antihistamines, stimulants, or other medications likely to affect the function of the nervous system (N=7)
Post-PCI
typical symptoms (dyspnoea、fatigue)
specific signs（Elevated jugular venous pressure、hepatojugular reflux、Pulmonary crackles、Peripheral oedema）
NT proBNP (ng/L) rule-in Age <50 yr: >450ng/L，Age 50–75 yr: >900，Age >75 yr: >1800
LVEF<50%
NT-proBNP and lncRNA-NRF were measured at intraprocedural and post-PCI at 4, 12, 24, and 72 h
exclude
include
HF (N=76)
Included in this study, discharged 4-5 days post-PCI
Abbreviations: AMI, acute myocardial infraction; HF, heart failure; NT-proBNP, N-terminal pro-brain natriuretic peptide;
 LVEF, left ventricular ejection fraction; PCI, percutaneous coronary intervention
